# A highlight on carbamazepine-induced adverse drug reactions in Saudi Arabia: a retrospective medical records-based study

**DOI:** 10.1007/s00210-023-02525-2

**Published:** 2023-05-18

**Authors:** Hatouf H. Sukkarieh, Ayesha A. Khokhar, Rami T. Bustami, Gulsan A. Karbani, Fatimah A. Alturki, Syed N. Alvi

**Affiliations:** 1 Department of Pharmacology, Alfaisal University, Riyadh, Kingdom of Saudi Arabia; 2 College of Medicine, Alfaisal University, Riyadh, Kingdom of Saudi Arabia; 3 Department of Operations & Project Management, Alfaisal University, Riyadh, Kingdom of Saudi Arabia; 4 Department of Genetic Counseling, Alfaisal University, Riyadh, Kingdom of Saudi Arabia; 5 Department of Genetic Counseling, King Abdulaziz Medical City, Riyadh, Kingdom of Saudi Arabia; 6 Department of Cell Biology, King Faisal Specialist Hospital & Research Centre, Riyadh, 11211 Kingdom of Saudi Arabia

**Keywords:** Carbamazepine, Gastrointestinal discomfort, Respiratory side effects, Systemic hypersensitivity

## Abstract

The link between human leukocyte antigen (HLA) alleles and carbamazepine-induced cutaneous, respiratory, and gastrointestinal adverse drug reactions (ADR) has created a window of opportunity for preventing certain forms of cutaneous adverse drug reactions (cADRs); however, there is not enough data to make pharmacogenomic recommendations that can be implemented globally. The aim of this study is to assess and document carbamazepine-induced adverse reactions among prescribed Saudi/non-Saudi patients. A retrospective chart review was performed for patients who received carbamazepine (CBZ) in the period between 2016 and 2020, in the Kingdom of Saudi Arabia. Data were gathered and descriptive statistical analyses were performed on the data for the study sample. Comparisons were made using the chi-square test or independent samples’ *t*-test. Statistical significance was considered at *p* < .05. All statistical analyses were performed using IBM SPSS 21.0 (Armonk, NY; IBM Corp). Results from multivariate logistic regression analyses showed that higher likelihood of carbamazepine-induced adverse reactions was significantly associated with younger age, OR = 0.82, 95% CI (0.74, 0.90); *p* < 0.001. Patients who were prescribed CBZ for reasons other than epilepsy or seizures were significantly more likely to develop carbamazepine-induced adverse reactions (epilepsy vs. other; OR = 0.63, *p* = 0.013; seizures vs. other; OR = 0.59, *p* = 0.018). Gender or medication duration were not related to carbamazepine-induced adverse reactions (*p* > 0.05). The findings of this study are comparable with those of other studies assessing carbamazepine-associated adverse reactions in children and adults. Recommendations include genetic prescreening, educating patients and parents on the possibility of adverse reactions, and routine laboratory monitoring.

## Introduction

Carbamazepine (CBZ) is a well-known antiepileptic drug (AED) that is used to treat various neurological conditions such as epilepsy, neuropathic pain, trigeminal neuralgia, and as a mood stabilizer for bipolar disorder (Fricke-Galindo and LLerena, A., Jung-Cook, H., & López-López, M. 2018). Even though CBZ is one of the best-tolerated AED, it is estimated that 70% of patients on AED therapy experience at least one adverse drug reaction (ADR) (Koliqi et al. 2015) which in turn compromise epilepsy management effectiveness (Fricke-Galindo and LLerena, A., Jung-Cook, H., & López-López, M. 2018). One of the ADR reported with CBZ administration is moderate to severe cutaneous reactions which has been shown to occur in 1–10% of patients taking CBZ, for example, on severe cutaneous reaction DRESS (drug reactions with eosinophilia and systemic symptoms), acute generalized exanthematous pustulosis (AGP) (Lin et al. 2018), and Stevens-Johnson syndrome (SJS)/toxic epidermal necrolysis (TEN). These adverse effects can be fatal, with mortality rates ranging from 10 to more than 30% (Fan et al. 2017). CBZ is also known to cause respiratory and gastrointestinal ADR such as wheezing, acute interstitial inflammation (Tolmie et al. 1983), pulmonary eosinophilia, asthma (Tolmie et al. 1983; Lee et al. 1981; Cullinan and Bower 1975), diarrhea, abdominal cramping and tenderness, hematochezia (Tuqan et al. 2018), lymphocytic colitis (Ianiro et al. 2012), and eosinophilic colitis (Eland et al. 1999; Atkinson et al. 2004; Anttila and Valtonen 1990, 1992).

CBZ-10,11-epoxide is involved in the process that underpins several of these ADR. CBZ is typically converted to CBZ-10,11-epoxide by the enzymes CYP3A4, CYP3A5, and CYP2C8 in the liver. Since of its tendency to generate covalent protein adducts (Bu et al. 2005), this reactive metabolite is of major importance because it is pharmacologically active and possibly toxic (Korinthenberg et al. 1994). Interindividual heterogeneity exists in CBZ-10,11-epoxide plasma levels, which range from 15 to 55% and 5 to 81% of CBZ concentrations in adults and children, respectively (Bertilsson and Tomson 1986). The skin is the primary tissue damaged in carbamazepine-induced cutaneous adverse drug reactions (cADRs), either through direct cellular damage caused by the drug or its metabolites or through an immune-mediated hypersensitivity process (Zaccara et al. 2007). CBZ is thought to bind directly to immunological receptors, notably T-cell receptors (TCR) and human leukocyte antigens (HLA), according to the studies (Yun et al. 2016). The strongest association between the HLA-B*15:02 allele and carbamazepine-induced SJS/TEN has been discovered (Fricke-Galindo and LLerena, A., Jung-Cook, H., & López-López, M. 2018) but nothing so far has been reported in the Kingdom of Saudi Arabia or the Gulf region. According to the current evidence, the risk correlation appears to be associated with a variety of carbamazepine-induced cutaneous adverse drug reaction (cADR) diseases in people of various ethnicities. The US Food and Drug Administration (FDA) suggested genetic testing before starting carbamazepine treatment for people with ancestors from regions where the HLA B*15:02 allele is found in December 2007 (Ferrell and McLeod 2008). Regulatory authorities in the UK, Singapore, Hong Kong, Thailand, and India soon followed with similar recommendations (Pan et al. 2017). In Taiwan, the Taiwan Food and Drug Administration (TFDA) added a new warning to address the genetic link in September 2008, and Taiwan’s National Health Insurance (NHI) funded the screening test for the HLA-B*15:02 allele in June 2010 (Pan et al. 2017).

ADR remain a common and major problem in healthcare (Gayford and Redpath 1969). Certainly, the link between HLA alleles and carbamazepine-induced cADRs has created a window of opportunity for preventing certain forms of ADR; however, there is not enough data to make pharmacogenomic recommendations that can be implemented globally. International agencies advise against using CBZ in people who have the HLA-B*15:02 and HLA-A*31:01 alleles, especially in vulnerable groups, and to use it only if the benefits outweigh the risks (Fricke-Galindo and LLerena, A., Jung-Cook, H., & López-López, M. 2018). The financial burden of ADR-related hospitalizations on global healthcare systems is significant. To not only reduce incidence rates and avoid episodes of disease and mortality, but also to enable physicians to better manage patient therapy, strategies to discover novel associated risk variables are required (Mullan et al. 2019). The aim of this study is to assess and document carbamazepine-induced adverse reactions among prescribed Saudi/non-Saudi patients.

## Materials and methods

A retrospective chart review was performed for patients who received carbamazepine in the period between 2016 and 2020, in the Kingdom of Saudi Arabia. Data were gathered on patient age, gender, nationality, reason for medication prescription, duration of medication usage, and variety of side effects including SJS, TEN, general skin eruption to drugs, diarrhea, runny nose, asthma, upper respiratory infection, limb swelling, skin reactions, and fever. The study has been approved by King Faisal Specialist Hospital and Research Centre; data source used were medical records and ADR were received from the adverse drug reaction reporting centre at the King Faisal Specialist Hospital and Research Centre. Inclusion criteria included all patients taking CBZ for the past 5 years while patients who were taking other AED were excluded. Data were uploaded and saved into an appropriately designed Excel spreadsheet and were processed in accordance with the best practices for raw data management to identify any inaccuracies or incompleteness before the statistical analyses. Data were compared against the possible minimum and maximum values of each variable and items with implausible values were flagged. A similar process was applied to demographic variables to identify any potential anomalies by running general frequency analyses. Descriptive statistical analyses were performed on the data for the study sample. Continuous variables were summarized using mean and standard deviation (SD), median, and IQR; and proportions were used for nominal and ordinal variables. A composite indicator of carbamazepine-induced adverse reactions was analyzed and compared by demographic and medication-related factors. Comparisons were made using the chi-square test or independent samples *t*-test. A logistic regression model was utilized to examine the independent effect of demographic and medication-related factors on the likelihood of developing carbamazepine-induced adverse reactions. Statistical significance was considered at *p* < 0.05. All statistical analyses were performed using IBM SPSS 21.0 (Armonk, NY; IBM Corp).

## Results

A total of 3071 patients were included with an average age of 30 years (SD = 16.4) and 52% females. Most patients were Saudi (97%). The distribution for reason for medication administration was as follows: seizure 38.8%, epilepsy 28.6%, and other reasons 32.6%. Average medication duration was 7.2 months (SD = 5.0).

The distribution of carbamazepine-induced adverse reactions was as follows: upper respiratory infection 2.9%, fever 2.4%, diarrhea 1.1%, asthma 1%, < 1% of patients developed general skin eruption to drug, runny nose, and limb swelling and none of the patients developed any toxic epidermal necrolysis (TEN) or skin reactions (Table 1).

**Table 1:** Baseline demographic and clinical characteristics of study patients. *n* = 3071

Characteristic	Value
Age (years) mean ± SD	30.0 ± 16.4
Gender *n* (%)	
Male	1603 (52.2%)
Female	1467 (47.8%)
Nationality *n* (%)	28.97 ± 5.32
Saudi	2989 (97.3%)
Non-Saudi	82 (2.7%)
Reason for medication administration *n* (%)	
Epilepsy	878 (28.6%)
Seizure	1191 (38.8%)
Other	1002 (32.6%)
Medication duration (months) mean ± SD	7.2 ± 5.0
Carbamazepine-induced adverse reactions	
Toxic epidermal necrolysis (TEN) *n* (%)	0 (0%)
General skin eruption to drugs *n* (%)	1 (< 1%)
Diarrhea *n* (%)	35 (1.1%)
Runny nose *n* (%)	1 (< 1%)
Asthma *n* (%)	32 (1.0%)
Upper respiratory infection *n* (%)	89 (2.9%)
Limb swelling *n* (%)	1 (< 1%)
Skin reactions *n* (%)	0 (0%)
Fever *n* (%)	74 (2.4%)
Composite outcome* *n* (%)	202 (6.6%)

*Includes any of the above listed carbamazepine-induced adverse reactions

Two hundred and two patients developed carbamazepine-induced adverse reactions (6.6%); 2.9% had upper respiratory infection, 2.4% fever, 1.1% diarrhea, 1% asthma, < 1% had runny nose or limb swelling, and none of the patients had toxic epidermal necrolysis or skin reactions. Patients developing carbamazepine-induced adverse reactions were compared with those who did not develop reactions in terms of their demographic and clinical characteristics (Table 2).

**Table 2:** Carbamazepine-induced adverse reactions by demographic clinical characteristic. *n* = 3071

Characteristic	Composite outcome—yes (202, 6.6%)	Composite outcome – no (2869, 93.4%)	*p*-value*
Age (years) mean ± SD	26.5 ± 18.4	30.2 ± 16.2	0.006
Gender *n* (%)			0.34
Male	112 (7.0%)	1491 (93.0%)	
Female	90 (6.1%)	1378 (93.9%)	
Nationality *n* (%)			0.24
Saudi	194 (6.5%)	2795 (93.5%)	
Non-Saudi	8 (9.8%)	74 (90.2%)	
Reason for medication admin *n* (%)			0.077
Epilepsy	55 (6.3%)	823 (93.7%)	
Seizure	67 (5.6%)	1124 (94.4%)	
Other	80 (8.0%)	922 (92.0%)	
Medication duration (months) mean ± SD	6.9 ± 4.7	7.2 ± 5.0	0.32

*Based on the *χ*^2^ test or *t*-test/Mann–Whitney *U* test

Results from multivariate logistic regression analyses (Table 3) showed that higher likelihood of carbamazepine-induced adverse reactions was significantly associated with younger age, OR = 0.82, 95% CI (0.74, 0.90); *p* < 0.001. Patients who were prescribed carbamazepine for reasons other than epilepsy or seizures were significantly more likely to develop carbamazepine-induced adverse reactions (epilepsy vs. other; OR = 0.63, *p* = 0.013; seizures vs. other; OR = 0.59, *p* = 0.018). Gender or medication duration were not related to carbamazepine-induced adverse reactions (*p* > 0.05).

**Table 3:** Multivariate logistic regression model for carbamazepine-induced adverse reactions. *n* = 3071

Factor	Mean or %	OR	95% CI	*p*-value
Age (per 10 years)	26.5	0.82	(0.74,0.90)	< 0.001
Gender				
Male	52.2%	1.15	(0.86,1.53)	0.35
Female	47.8%	1.00	Ref	
Reason for medication admin				
Epilepsy	28.6%	0.63	(0.43,0.91)	0.013
Seizure	38.8%	0.59	(0.41,0.83)	0.018
Other	32.6%	1.00	Ref	

Odds ratio was reported as adjusted OR

*OR*, odds ratio; *CI*, confidence interval

## Discussion

Carbamazepine remains a first-line drug for treatment of epilepsy in children and adults. Results in this study show that two hundred and two patients developed carbamazepine-induced adverse reactions (6.6%); 2.9% had upper respiratory infection. This is consistent with several other studies, which show the involvement of the immune system post taking CBZ, usually due to a transient decline in Immunoglobulin A (IgA). There are several reports on CBZ-associated pulmonary complications, including interstitial pneumonitis. These complications were mainly described in adults and are considered rare side effects. A few studies reported the occurrence of CBZ associated interstitial pneumonitis in pediatric patients too (Gonçalves et al. 2012; Swert et al. 1984). Moreover, there has been possible links between CBZ to known immune-related effects, which can result in a high fever (Sheridan et al. 1982).

Our results show that 2.4% of patients experienced high fever. On the other hand, gastrointestinal (GI) discomfort has been shown in patients taking CBZ (Sheridan et al. 1982). In our study, 1.1% of patients experienced severe diarrhea. This is considered among the most common side effects of AED in general and especially CBZ. These GI adverse effects lead to the discontinuation or irregular consumption of CBZ. Studies have shown that the incidence of diarrhea increases among patients receiving more than one AED, such as valproic acid and gabapentin. Reported GI adverse effects linked to AED are mainly heartburn, nausea, constipation, vomiting, diarrhea, and dysphagia. Nausea and vomiting were significantly higher in patients receiving monotherapy. GI adverse effects can have a significant effect on the drug absorption and overall utilization which will reflect on the efficacy of the therapy and increase the number of epileptic attacks (Jahromi et al. 2011). Our results showed < 1% limb swelling. This is consistent with published data showing that CBZ is associated with severe systemic hypersensitivity reaction, known as carbamazepine hypersensitivity syndrome (CHS). CHS consists of mainly three symptoms: fever, lymphadenopathy, and rash, hence referred to as pseudolymphoma syndrome (Mesec et al. 1999). There are reports of skin biopsy showing vasculitis of small vessels with perivascular infiltrates of lymphocytes, monocytes, and macrophages post-CBZ administration. The findings are compatible with experiencing an allergic reaction (Harats and Shalit 1987).

CBZ has been shown to be a cause of cutaneous vasculitis. This is extremely uncommon. Our study compared patients who developed carbamazepine-induced adverse reactions with those who did not develop reactions in terms of their demographic and clinical characteristics (Table 2).

In 1987, age restriction was lifted for the use of carbamazepine. Since then, carbamazepine gained approval for use in patients of all ages, which led to the rapid growth in prescribing this drug. Our results showed higher likelihood of carbamazepine-induced adverse reactions in younger patients OR = 0.82, 95% CI (0.74, 0.90); *p* < 0.001. Protein binding may be decreased in infants, resulting in a greater proportion of free (active) drug. However, in this study, we did not include any pediatric patients. Our finding was not consistent with studies reporting that elderly patient experience more adverse effects than young adults (Hockings et al. 1986; Pellock 1987).

Patients who were prescribed CBZ for reasons other than epilepsy or seizures were significantly more likely to develop carbamazepine-induced adverse reactions (epilepsy vs. other; OR = 0.63, *p* = 0.013; seizures vs. other; OR = 0.59, *p* = 0.018). We were not able to find any evidence in the literature regarding these findings. Gender or medication duration were not related to carbamazepine-induced adverse reactions (*p* > 0.05).

The strength of our study lies in the number of patients; also, this study is one of the only few as reporting of serious ADR in the Gulf region and in Kingdom of Saudi Arabia; hence, no genetic screening is being done before the prescription of CBZ. We are aiming to shift from traditional medicine to personalized medicine. Limitation is that ADR are required to be reported more often in a systematic way since there is an under-reporting due to not having a system in place at King Faisal Specialist Hospital and Research Centre.

## Conclusion

The findings of this study are comparable with those of other studies assessing carbamazepine-associated adverse reactions in children and adults. Recommendations for carbamazepine therapy include education of patients and parents on the nature and likelihood of possible serious adverse reactions and routine monitoring to detect laboratory abnormalities. We should also increase the awareness of reporting adverse drug reactions among physicians as they are the first-line encounters of the patients.

## Data Availability

Data were obtained from King Faisal Specialist Hospital and Research Centre. The authors confirm that the data supporting the findings of this study are available within the article. Raw data that support the findings of this study are de-identified and securely saved in Microsoft Excel. The data are available from the corresponding author on reasonable request.
